# Approximate Bayesian computation schemes for parameter inference of discrete stochastic models using simulated likelihood density

**DOI:** 10.1186/1471-2105-15-S12-S3

**Published:** 2014-11-06

**Authors:** Qianqian Wu, Kate Smith-Miles, Tianhai Tian

**Affiliations:** 1 School of Mathematical Sciences, Monash University, Melbourne, Australia

## Abstract

**Background:**

Mathematical modeling is an important tool in systems biology to study the dynamic property of complex biological systems. However, one of the major challenges in systems biology is how to infer unknown parameters in mathematical models based on the experimental data sets, in particular, when the data are sparse and the regulatory network is stochastic.

**Results:**

To address this issue, this work proposed a new algorithm to estimate parameters in stochastic models using simulated likelihood density in the framework of approximate Bayesian computation. Two stochastic models were used to demonstrate the efficiency and effectiveness of the proposed method. In addition, we designed another algorithm based on a novel objective function to measure the accuracy of stochastic simulations.

**Conclusions:**

Simulation results suggest that the usage of simulated likelihood density improves the accuracy of estimates substantially. When the error is measured at each observation time point individually, the estimated parameters have better accuracy than those obtained by a published method in which the error is measured using simulations over the entire observation time period.

## Background

In recent years, quantitative methods have become increasingly important for studying complex biological systems. To build a mathematical model of a complex system, two main procedures are commonly conducted [1]. The first step is to determine the elements of the network and regulatory relationships between the elements. In the second step, we need to infer the model parameters according to experimental data. Since biological experiments are time-consuming and expensive, normally experimental data are often scarce and incomplete compared with the number of unknown model parameters. In addition, the likelihood surfaces of large models are complex. The calibration of these unknown parameters within a model structure is one of the key issues in systems biology [2]. The analysis of such dynamical systems therefore requires new, effective and sophisticated inference methods.

During the last decade, several approaches have been developed for estimating unknown parameters: namely, optimization methods and Bayesian inference methods. Aiming at minimizing an objective function, optimization methods start with an initial guess, and then search in a directed manner within the parameter space [3,4]. The objective function is usually defined by the discrepancy between the simulated outputs of the model and sets of experimental data. Recently, the objective function has been extended to a continuous approach by considering simulation over the whole time period [5] and a multi-scale approach by including multiple types of experimental information [6]. Several types of optimization methods can be found in the literature, among which two major types are called gradient-based optimization methods and evolutionary-based optimization methods. Based on these two basic approaches, various techniques such as simulated annealing [7]. linear and non-linear least-squares fitting [8], genetic algorithms [9] and evolutionary computation [10,11] have been attempted to build computational biology models. Using optimization methods, the inferred set of parameters produces the best fit between simulations and experimental data [12,13]. which have been successfully applied for biological systems, however, there are still some limitations with these methods such as the problem of high computational cost when significant noise exists in the system. To address these issues, deterministic and stochastic global optimization methods have been explored [14].

When modeling biological systems where molecular species are present in low copy numbers, measurement noise and intrinsic noise play a substantial role [15], which is a major obstacle for modeling. Bayesian inference methods have been used to tackle such difficulties by extracting useful information from noise data [16]. The main advantage of Bayesian inference is that it is able to infer the whole probability distributions of parameters by updating probability estimates using Bayes' Rule, rather than just a point estimate from optimization methods. Also. Bayesian methods are more robust than using other methods when they are applied to estimate stochastic systems, which is not that obvious for modeling of deterministic systems [17]. Developments have taken place during the last 20 years and recent advances in Bayesian computation including Markov chain Monte Carlo (MCMC) techniques and sequential Monte Carlo (SMC) methods have been successfully applied to biological systems [18,19].

For the case of parameter estimation when likelihoods are analytically or computationally intractable, approximate Bayesian computation (ABC) methods have been applied successfully [20,21]. ABC algorithms provide stable parameter estimates and are also relatively computationally efficient, therefore, they have been treated as substantial techniques for solving inference problems of various types of models that were intractable only a few years ago [19]. In ABC. the evaluation of the likelihood is replaced by a simulation-based procedure using the comparison between the observed data and simulated data [22]. Recently, a semi-automatic method has been proposed to construct the summary statistics for ABC [23]. These methods have been applied in a diverse range of fields such as molecular genetics, epidemiology and evolutionary biology etc. [24-26].

Despite substantial progress in the application of ABC to deterministic models, the development of inference methods for stochastic models is still at the very early stage. Compared with deterministic models, there are a number of open problems in the inference of stochastic models. For example, recent work proposed ABC to infer unknown parameters in stochastic differential equation models [27]. Our recent computational tests [28] showed the advantages and disadvantages of a published ABC algorithm for stochastic chemical reaction systems in [17]. In this work, we propose two novel algorithms to improve the performance of ABC algorithms using the simulated likelihood density.

## Results and discussion

### The first test system with four reactions

We first examine the accuracy of our proposed methods using a simple model of four chemical reactions [29]. The first reaction is the decay of molecule *S*_1_. Then two molecules *S*_1 _form a dimer *S*_2 _in the second reaction; and this dimerization process is reversible, which is represented by the third reaction. The last reaction in the system is a conversion reaction from molecule *S*_2 _to its product *S*_3_. All these four reactions are given by

$$\begin{array}{ccc} S_{1} & \overset{c_{1}}{\to } & \left(\right),\\ S_{1}+S_{1} & \overset{c_{2}}{\to } & S_{2},\\ S_{2} & \overset{c_{3}}{\to } & S_{1}+S_{1},\\ S_{2} & \overset{c_{4}}{\to } & S_{3}. \end{array}$$

**Figure 1:**
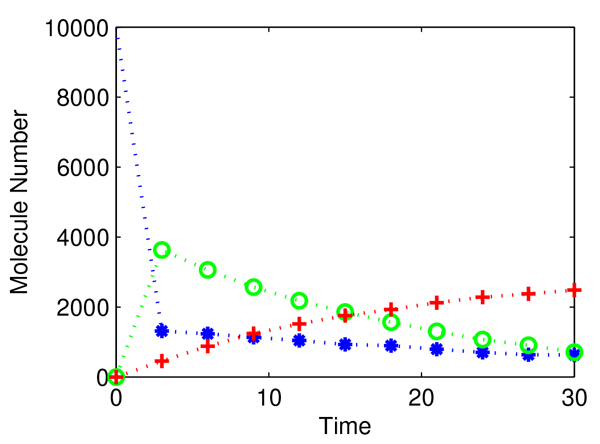
**Simulated experimental data for system dynamics in a time length of 30 with step size Δ*t *of 3**. Blue star for *S*_1_, green circle for *S*_2_, and red cross for *S*_3_.

We start with an initial condition with **S **= (10000, 0,0) and rate constants of **c **= (0.1,0.002,0.5,0.04), which is termed as the exact rate constants in this test. The stochastic simulation algorithm (SSA) was used to simulate the stochastic system [30]. A single trajectory for this model during a period of *T *= 30 in a step size of Δ*t *= 3 is presented in Figure 1.

**Figure 2:**
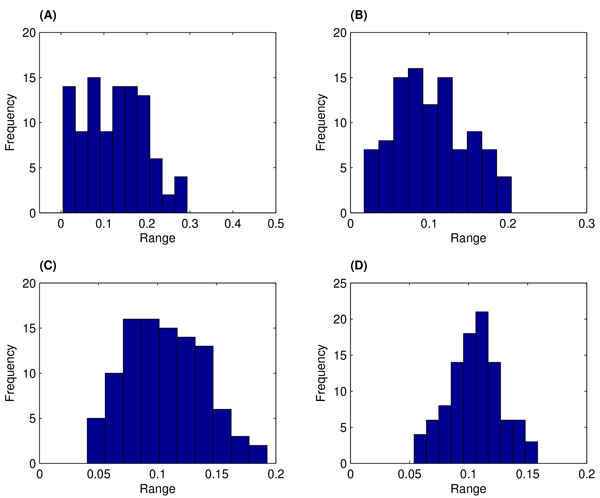
**Probabilistic distributions of the estimated rate constant of *c*_1 _over four iterations using algorithm 1**. (A): Iteration 2; (B): 3; (C): 4; (D): 5.

When applying the algorithms in the Method section to estimate model parameters, we assumed the prior distribution for each estimated parameter follows a uniform distribution π(*θ*) ~ U(0, *A*). For rate constants *c*_1 _~ *c*_4_, the values of *A *are (0.5, 0.005, 1, 0.1). Figure 2 shows probabilistic distributions of the estimated rate constant of *c*_1 _over iterations (2 ~ 5). In this test, we have the step size Δ*t *= 3 and simulation number *B*_k _= 10. Figure 2 suggests that the probabilistic distribution starts from nearly a uniform distribution in the second iteration (Figure 2A) and gradually converges to a normalized-like distribution with a mean value that is close to the exact rate constant.

There are two tolerance values in the proposed algorithms, namely *α *for the discrepancy in step 2.c and *∈_k _*for the fitness error in step 2.d. In the following tests, we considered two strategies: the value of a is a constant [31] or its value varies over iterations. To examine the factors that influence the convergence rate of particles over iterations, we calculated the mean count number for each iteration, which is the averaged number of counts for accepting all simulated estimation of parameter sets. The averaged error is defined by the sum of relative errors of each rate constant for each iteration. Table 1 displays the performances of the tests under three schemes which used fixed discrepancy tolerance *α *= 0.1, 0.05 or varying values of *α*. In each case, we used the same values of *∈_k _*for the fitness tolerance. The value of *α *in the varying *α *strategy equals the value of *∈_k_*, namely *α_k _*= *∈_k_*.

**Table 1:** Comparison of averaged error and mean count number for estimated rate constants over five iterations using algorithms 1 and 2 with simulation number of 10 for system 1.

Δ*t*	*α\k*		1	2	3	4	5
**Algorithm 1**							

3	0.1	MN	15.41	7.21	7.36	8.21	10.05
		AE	0.7668	0.7294	0.7073	0.7832	0.6173
	0.05	MN	175.72	30.66	24.47	28.22	26.5
		AE	0.6120	0.5036	0.5521	0.7175	0.6132
	vary	MN	46.46	25.07	22.76	30.09	88.56
		AE	0.7669	0.5306	0.6780	0.5858	0.5945
5	0.1	MN	26.96	10.47	9.07	11.18	13.19
		AE	0.7107	0.5607	0.5366	0.4693	0.4853
	0.05	MN	130.64	27.38	25.42	35.36	35.79
		AE	0.5826	0.6495	0.4260	0.7548	0.4139
	vary	MN	141.97	30.28	53.47	127.16	2911.58
		AE	0.5587	0.4793	0.5416	0.5960	0.5375

**Algorithm 2**							

3	0.05	MN	467.61	52.34	41.08	69.17	195.69
		AE	0.5834	0.6091	0.4867	0.4995	0.4402
	vary	MN	100.26	32.04	24.78	80.15	1793.64
		AE	0.7132	0.6657	0.6305	0.6705	0.4833
5	0.05	MN	333.17	24.26	32.85	21.11	21.84
		AE	0.5962	0.5340	0.5761	0.4983	0.5518
	vary	MN	243.78	22.6	31.29	34.6	70.25
		AE	0.6565	0.6035	0.5759	0.5488	0.4263
							

Tests are experimented under different strategies of discrepancy tolerance such as α = 0.1, 0.05 or varies over iterations (AE:Averaged Error; MN: Mean count Number).

In these performances, we used *∈_k _*= (0.07, 0.06, 0.055, 0.05, 0.045) and (0.05, 0.045, 0.04, 0.035, 0.03) for algorithm 1 with step sizes Δ*t *= 3 and 5, respectively. For algorithm 2, these values are *∈_k _*= (0.095, 0.08, 065, 0.05, 0.04) and (0.059, 0.055, 0.05, 0.045, 0.04). An interesting observation is that the values of mean count number are very large in the first iteration, then decrease sharply and stay within a value stably. We have a detailed test of using different values of the fitness tolerance *∈_k _*and found that when using step size of Δ*t *= 3, mean count number stays at one if *∈_k _*≥ 0.1; but it starts to increase sharply to a large number if *∈_k _*< 0.1. The observation numbers using a step size of Δ*t *= 3 is 10 and the maximum error that can incur calculated from step 2.d is 0.1 with one hundred particles. Similarly, this critical *∈_k _*value is 0.06 for a step size of Δ*t *= 5.

Meanwhile all averaged errors have a decreasing trend over iterations. Looking at different cases with various values of discrepancy tolerance *α *, it is also observed that using *α *= 0.1 results in more discrepancies of the estimated parameters on average than the other two cases, in particular, than the case *α *= 0.05. Thus in our following tests, we just concentrate on the cases of *α *= 0.05 and varying *α*. In addition, we observe that by taking *α *= 0.05 for the case with step size of Δ*t *= 3, it leads to more accurate approximation since *α *= 0. 05 is less than most values of *α *in the case of varying values of α. It is consistent with the cases of a step size of Δ*t *= 5 in which little differences can be found comparing strategies using *α *= 0.05 and *α *= *∈_k _*since the values of *∈_k _*are quite close to 0.05. In the case of varying values of α, a small value of *ε*_5 _leads to a small value of *α*_5_, which results in a substantial increase in mean count number. However, this large mean count number does not necessary bring more accurate estimated parameters. With these findings, we simulated results using *α *= 0.05 and *α *= *∈ *only for algorithm 2. Consistent results are obtained using algorithm 2. Moreover, results obtained using algorithm 2 is more accurate than those from algorithm 1.

### The second test system with eight reactions

Although numerical results of the first test system are promising regarding the accuracy, that system has only four reactions. Thus the second test system, namely a prokaryotic auto-regulatory gene network, includes more reactions. This network involves both transcriptional and translational processes of a particular gene. In addition, dimers of the protein suppress its own gene transcription by binding to a regulatory region upstream of the gene [32-34]. This gene regulatory network consists of eight chemical reactions which are given below:

R1:DNA+P2→c1DNA·P2,R2:DNA·P2→c2DNA+P2,R3:DNA→c3DNA+mRNA,R4:mRNA→c40,R5:2P→c5P2,R6:P2→c62P,R7:mRNA→c7mRNA+P2,R8:P→c80.

This gene network includes five species, namely DNA, messenger RNA, protein product, dimeric protein, and the compound formed by dimeric protein binding to the DNA promoter site, which are denoted by DNA, mRNA, P, P_2 _and DNA · P_2_, respectively. In this network, the first two reactions R_1 _and R_2 _are reversible reactions for dimeric protein binding to the DNA promoter site. Reactions R_3 _and R_7 _are transcriptional and translation processes for producing mRNA and protein, respectively. Reactions R_5 _and R_6 _represent the interchange between protein P and dimeric protein P_2. _The system ends up with a degradation process of protein P [32].

To apply our algorithms, we start up with an initial condition of molecular copy number

$$\left(\text{DNA},\text{ mRNA},\text{ P},\;{\text{P}}_{\text{2}},\text{ DNA}\cdot {\text{P}}_{\text{2}}\right)\;=\;\left(\text{1}0,\text{1}00,\text{1}00,\text{ 8}00,\text{1}00\right).$$

**Figure 3:**
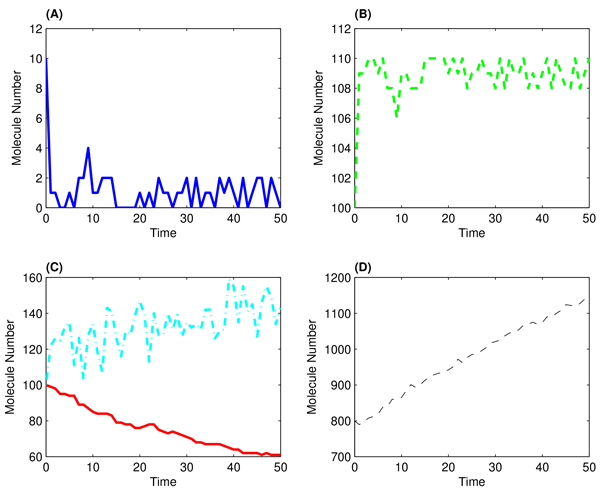
**Simulated molecular numbers for system 2 in a time length of 50 with step size Δ*t *of 1**. (a): DNA numbers; (B): numbers of DNA · P_2_; (C): Red line for the numbers of mRNA black and cyan dash-dotted line for the numbers of P; (D): numbers of P_2_.

In addition, the following reaction rate constants

$$\left(c_{\text{1}},...,c_{\text{8}}\right)\;=\;\left(0.\text{1},\;0.\text{7},\;0.\text{35},\;0.0\text{1},\;0.\text{1},\;0.\text{9},\;0.\text{2},\;0.0\text{1}\right)$$

are used as the exact rate constants to generate a simulation for each molecular species during a period of *T *= 50 in a step size of Δ*t *= 1 and results are presented by Figure 3. This simulated dataset is used as observation data for inferring the rate constants.

The prior distribution of each parameter follows a uniform distribution *π*(*θ*) ~ *U*(0, *B*). For rate constants *c*_1 _~ *c*_8_, the values of *B *are (0.5,2,1,0.1,0.5, 5,1,0.1). The proposed two algorithms were implemented over five iterations and each iteration contains 100 particles. We choose step sizes Δ*t *= 2 or 5 and the number of stochastic simulation *B_k _*= 10.

**Figure 4:**
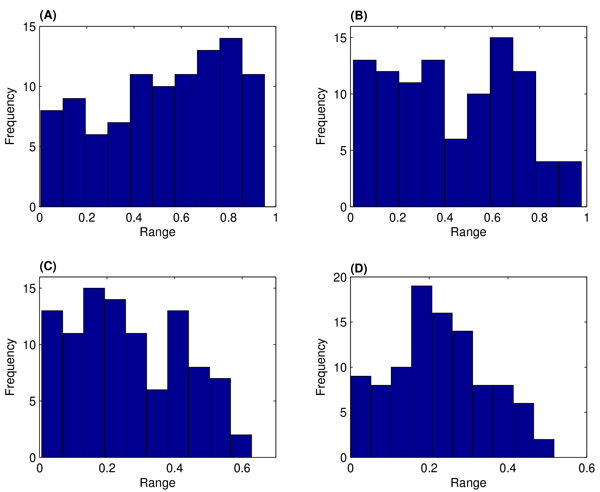
Probabilistic distributions of the estimated rate constant *c*_7 _over four iterations using algorithm **1**. (A):Iteration 2; (B): 3: (C):4; (D):5.

Figure 4 gives the probabilistic distribution of the estimated rate constant *c*_7 _over 2nd ~ 5th iterations. The distribution of the first iteration is close to the uniform distribution, and this is not presented. Since the second iteration, the estimated rate constant begins to accumulate around the exact value *c*_7 _= 0.2. At the last iteration, the probability in Figure 4D shows a normalized-like distribution. Compared with the results of system 1 in Figure 2, the convergence rate of the parameter distribution of system 2 is slower. Our numerical results suggested that this convergence rate depends on the strategy of choosing the values of discrepancy tolerance *α*.

To analyze the factors that influence the convergence property of estimates, the mean count number as well as the averaged error for each iteration *k *are obtained. Results are presented in Table 2. Using algorithm 1 and 2, we tested for step sizes of Δ*t *= 2 and Δ*t *= 5. Since the errors of estimates obtained using a fixed value of *α *= 0.1 are always larger than those obtained by *a *= 0.05, we only tested with the cases of a fixed value *α *= 0.05 and varying values of *α*. For algorithm 1, we tested two cases for the varying values of discrepancy tolerance *α*. In the first test, the values are *∈_k _*= (0.21,0.2,0.19,0.18,0.175) and *α *= *∈_k _*for varying values of *α*, which is the case "Same *∈_k_*" in Table 2. The values of *∈_k _*are also applied to the case of a fixed value *α *= 0.05. In this case, the averaged count number of varying *α* is much smaller than that of a fixed value of α. Thus we further decreased the value of *α* to (0.15,0.125,0.1,0.075,0.07), which is the case "Diff. *∈_k_*" in Table 2. In this case, the mean count numbers are similar to those using a fixed *α*. Numerical results suggested that the strategy of using a fixed value of *α *generates estimates with better accuracy than the strategies of using varying *α *values, even when the computing time of the varying *α *strategy is larger than that of the fixed *α *strategy.

**Table 2:** Comparison of averaged error and mean count number for estimated rate constants of system 2 using algorithms 1 and 2.

Δ*t*	*α\k*		1	2	3	4	5
**Algorithm 1**							

2	0.05	MN	18.29	7.53	9.8	12.7	14.23
		AE	4.6211	4.4179	4.7138	4.2188	3.8119
	Same ***∈_k_***	MN	2.69	2.07	2.16	1.93	1.93
		AE	4.7006	4.9603	4.8841	4.6833	4.7298
	Diff. ***∈_k_***	MN	15.26	7.85	8.78	13.06	12.28
		AE	4.8295	4.5322	5.0418	4.7346	4.6069
5	0.05	MN	9.69	3.48	3.12	58.2	74.07
		AE	4.1076	4.3243	4.1868	3.5311	3.5194
	Same ***∈_k_***	MN	2.34	2.31	2.42	16.9	11.38
		AE	4.9862	4.7669	4.6716	3.8873	4.0017
	Diff. ***∈_k_***	MN	25.72	8.14	10.45	25.8	174.88
		AE	4.0461	3.9583	3.7474	3.5655	3.6951

**Algorithm 2**							

2	0.05	MN	89.7	19.75	17.8	40.42	69.52
		AE	4.0540	4.1339	4.1376	3.9696	3.9009
	Same ***∈_k_***	MN	2.52	3.85	3.55	3.82	3.84
		AE	5.0456	4.6069	4.3666	4.5876	3.8958
	Diff. ***∈_k_***	MN	197.49	15.05	22.09	36.85	94.24
		AE	3.8712	3.7934	4.3158	3.6485	3.5989
5	0.05	MN	138.14	30.52	46.66	98.87	377.66
		AE	4.0258	3.7218	3.8258	3.8445	3.9205
	Same ***∈_k_***	MN	21.67	11.34	11.17	26.65	59.64
		AE	4.0545	3.5715	4.1910	3.7252	3.8667
	Diff. ***∈_k_***	MN	185.54	28.39	33.81	89.81	846.61
		AE	3.7810	3.6694	3.6939	3.9806	3.8515

Three strategies are used to choose the discrepancy tolerance *α*: a fixed value of *α*= 0.05; varying *α*values; and *α= ∈_k_*denoted as same *∈_k_*); varying *α*values that are smaller than *∈_k _*(denoted as diff. *∈_k_*).(AE:Averaged Error; MN: Mean count Number).

For algorithm 2, we carried out similar tests. In the first case, we set *∈_k _*=(0.24,0.23,0.22,0.21,0.2), which is applied to the strategy of fixing *α* = 0.05 and varying *α* with *α*= *∈_k _*that is the case "Same *∈_k_*" in Table 2. Again, the averaged count numbers of varying α strategy are much smaller than those using a fixed *α*. Thus we decreased the value to (0.095,0.09,0.085,0.08, 0.075), which is the case "Diff. *∈_k_*" in Table 2; However, the averaged count numbers in the "Diff. *∈_k_*" case are similar to those of the previous two strategies, namely a fixed a and "Same *∈_k_*". For algorithm 2, Table 2 suggests that the varying *α* strategy generates estimates that are more accurate than those obtained from the fixed *α *strategy. However, the best estimates in Table 2 are obtained using algorithm 1 and fixed *α *strategy.

## Conclusions

To uncover the information of biological systems, we proposed two algorithms for the inference of unknown parameters in complex stochastic models for chemical reaction systems. Algorithm 1 is in the framework of ABC SMC and uses transitional density based on the simulations over two consecutive observation time points. Algorithm 2 generates simulations of the whole time interval but differs from the published method in the error finding steps by comparing errors of simulated data to experimental data at each time point. The proposed new algorithms impose stricter criteria to measure the simulation error. Using two chemical reaction systems as the test problems, we examined the accuracy and efficiency of proposed new algorithms. Based on the results of two algorithms for system 1, we discovered that taking smaller values of discrepancy tolerance *α* will result in more accurate estimates of unknown model parameters. This conclusion is confirmed by the second system that we tested under different conditions. Numerical results suggested that the proposed new algorithms are promising methods to infer parameters in high-dimensional and complex biological system models and have better accuracy compared with the results of the published method [28]. The encouraging result is that new algorithms do not need more computing time to achieve such accuracy. Our computational tests showed that the selection for the value of fitness tolerance is a key step in the success of ABC algorithms. The advantage of the population Monte-Carlo methods is the ability to reduce the fitness tolerance gradually over populations. Generally, a smaller value of fitness tolerance will lead to a larger number of iteration count and consequently larger computing time. For deterministic inference problems, a smaller value of fitness tolerance normally will generate estimates with better accuracy. However, for stochastic models, this conclusion is not always true. In addition to the fitness tolerance, our numerical results suggested that other factors, such as the simulation algorithm for chemical reaction systems and the strategy of discrepancy tolerance, also have influences on the accuracy of estimates. Thus more skilled approaches, such as the adaptive selection process for the fitness tolerance, should be considered to improve the performance of ABC algorithms.

In this work, we used the SSA to simulate chemical reaction systems [30]. This approach may be appropriate when the biological system is not large. In fact, for the two biological systems discussed in this work, the computing time of inference is still very large. To reduce the computing time, more effective methods should be used to simulate the biological systems, such as the *τ*-leap methods [35] and multi-scale simulation methods [36,37]. Another alternative approach is to use parallel computing to reduce the heavy computing loads. All these issues are potential topics for future research work.

## Methods

### ABC SMC algorithm

ABC algorithms bypass the requirement for evaluating likelihood functions directly in order to obtain the posterior distributions of unknown parameters. Instead, ABC methods simulate the model with given parameters, compare the observed and simulated data, and then accept or reject the particular parameters based on the error of simulation data. Thus there are three key steps in the implementations of ABC algorithms. The first step is the generation of a sample of parameters θ* from the prior distribution of parameters or from other distributions that are determined in ABC algorithms. The second step is to define distance function d(**X, Y**) between the simulated data **X **and experimental observation data **Y**. Finally, a tolerance value is needed as a selection criterion to accept or reject the sampled parameter θ*. Based on the generic form of ABC algorithm [17], a number of methods have been developed including ABC rejection sampler and ABC MCMC [38,39]. The ABC rejection algorithm is one of the basic ABC algorithm that may result in long computing time when a badly prior distribution that is far away from posterior distribution is chosen. ABC MCMC introduces a concept of acceptance probability during the decision making step which saves computing time. However, this may result in getting stuck in the regions of low probability for the chain and we may never be able to get a good approximation. To tackle these challenges, the idea of particle filtering has been introduced. Instead of having one parameter vector at a time, we sample from a pool of parameter sets simultaneously and treat each parameter vector as a particle. The algorithm starts from sampling a pool of *N *particles for parameter vector ***θ ***through prior distribution *π*(*θ*). The sampled particle candidates $\left(\theta_{1}^{*},\cdots \;,\theta_{N}^{*}\right)$ will be chosen randomly from the pool and we will assign each particle a corresponding weight *ω *to be considered as the sampling probability. A perturbation and filtering process following through a transition kernel *q*(·|θ*) finds the particles θ**. Similarly with θ**, data **Y **can be simulated and compared with experimental data **X **to further fulfil the requirements for estimating posterior distribution.

The basic form of algorithm described above is as follows [19]:

Algorithm: ABC SMC

1. Define the threshold values $\in_{1},\cdots \;,\in_{K}$, start with iteration *k *= 1.

2. Set the particle indicator *i *= 1.

3. If *k *= 1, sample θ^* ^from the proposed prior distribution *π*(*θ*). Generate a candidate data set *D*(*_b_*)(θ*) *B_k _*times and calculate the value of *b_k_*(θ*), where *D*(*_b_*) ~ *p*(*D*|*θ*) for any fixed parameter *θ*,

(1) $$b_{k}(\theta^{*})=\overset{B_{k}}{\underset{b=1}{\sum }}1(d(D_{0},D_{\left(b\right)}(\theta^{*}))\leq \in_{k})$$

and *D*_0_ is the experimental data set.

If *k *> 1, sample *θ *from the previous population $\left\{\theta_{k-1}^{i}\right\}$ with weights *w_k−1 _*and perturb the particle to obtain θ^* ^using a kernel function $K_{k}$.

If *π*(θ*) = 0 or *b_k_*(θ*) = 0, return to the beginning of step 3.

4. Set $\theta_{k}^{i}=\theta^{*}$ and determine the weight for each estimated particles $\theta_{k}^{i}$,

$$w_{k}^{\left(i\right)}=\left\{\begin{array}{cc} b_{k}\left(\theta_{k}^{i}\right) & \text{if}\;\;k=1;\\ \frac{\pi \left(\theta_{k}^{i}\right)b_{k}\left(\theta_{k}^{i}\right)}{{\sum_{j=1}^{N}K}_{k}\left(\theta_{k-1}^{j},\theta_{k}^{i}\right)} & \text{if}\;\;k>1. \end{array}\right)$$

If *i *<*N*, update *i *= *i *+ 1 and return to step 3.

5. Normalize the weights $w_{k}^{\left(i\right)}$ If *k *<*K*, update *k *= *k *+1 and go back to step 2.

A number of algorithms have been developed using the particle filtering technique, such as the partial rejection control, population Monte-Carlo and SMC. Each of them differs in the formation of weight *w *and the transition kernels.

### ABC using simulated likelihood density

ABC SMC method uses the simulation over the entire time period to measure the fitness to experimental data, which is consistent to the approaches used for deterministic models [17]. For stochastic models, the widely used approach is treating transitional density as the likelihood function [40,41]. Based on a sequence of n + 1 observations **X **= [*X*_0_, *X*_1; _· · ·, *X*_n_] at time points [*t*_0_, *t*_1; _· · ·, *t*_n_], for a given parameter set *θ *the joint transitional density is defined as

(2) $$f_{0}\left[\left(t_{0},X_{0}\right)|\theta \right]\overset{n}{\underset{i=1}{\prod }}f\left[\left(t_{i},X_{i}\right)\left(t_{i-1},X_{i-1}\right),\cdots \;,\left(t_{0},X_{0}\right);\theta \right],$$

where *f*_0_[·] is the density of initial state, and

(3) $$f\left[\left(t_{i},X_{i}\right)|\left(t_{i-1},X_{i-1}\right),\cdots \;,\left(t_{0},X_{0}\right);\theta \right]$$

is the transitional density starting from (*t_i−1_, X_i −1_*) and evolving to (*t_i_, X_i_*). When the process *X *is Markov, the density (3) is simplified as

(4) $$f\left[\left(t_{i},X_{i}\right)|\left(t_{i-1},X_{i-1}\right);\theta \right].$$

In the simulated likelihood density (SLD) methods, this transitional density is approximated by that obtained from a large number of simulations.

Based on the discrete nature of biochemical reactions with low molecular numbers, it was proposed to use the frequency distribution of simulated molecular numbers to calculate the transitional density [31]. The frequency distribution is evaluated by

$$F\left[X=X_{l}\right]=\frac{1}{B_{k}}\overset{B_{k}}{\underset{m=1}{\sum }}\left[1-\delta \left(X_{l},X_{ml}\right)\right]$$

using *B_k _*simulations with the simulated state *X_ml_*. Here the function *δ*(*x*) is defined by

$$\delta \left(X_{l},X_{ml}\right)=\left\{\begin{array}{cc} 0 & \text{if}\;\;d\left(X_{l},X_{ml}\right)<\alpha X_{l};\\ 1 & \text{else}, \end{array}\right)$$

where *d*(*x, y*) is a distance measure between *x *and *y*.

Here we propose a new algorithm that uses the simulated transitional density function as the objective function. Unlike ABC SMC algorithm [17], the new method considers the transitional density function from *t_i −1 _*to *t_i _*only at each step. Based on the framework of ABC SMC, the new algorithm using transitional density is proposed as follows.

ABC SLD algorithm 1

1. Given data **X **and any assumed prior distribution *π*(*θ*), define a set of threshold values *∈*_1_, · · ·, *∈_K_*.

2. For iteration *k *= 1,

(a) Set the particle indicator *i *= 1, sample θ* ~ *π*(*θ*).

(b) For time step *l *= 1,2, · · ·, *n*, use initial condition **X***_l − 1 _*and parameter θ^* ^to generate data **Y **at *t_l _*for *B_k _*times.

(c) For *m *= 1, ·· ·, *B_k_*, calculate the value of discrepancy and test for

(5) $$d\left(X_{l},{Y_{m}}_{1}\right)\;\leq \alpha {\text{X}}_{l},$$

where *α *is a defined constant.

If it is true, let *β_ml _*(θ*) = 0, otherwise it is one. Then determine

(6) $$b_{l}\left(\theta^{*}\right)=\overset{B_{k}}{\underset{m=1}{\sum }}\beta_{ml}\left(\theta^{*}\right).$$

(d) Calculate

(7) $$\in =\overset{m}{\underset{l=1}{\sum }}\frac{1}{B_{k}}\left(B_{k}-b_{l}\left(\theta^{*}\right)\right).$$

If *∈ *<*∈_k_*, update $\theta_{i}^{k}=\theta^{*}$ and move to the next particle *i *= *i *+1.

(e) Assign weight $w_{i}^{k}=\frac{1}{N}$ for each particle.

3. Determine the variance for the particles in the first iteration

$$\sigma_{1}=\sqrt{\mathrm{var}\left(\theta_{\left\{1:N\right\}}^{1}\right)}$$

4. For iteration *k *= 2, · · ·, *K*

(a) Start with *i *= 1, Sample $\theta^{*}\sim \theta_{i:N}^{k-1}$ using the calculated weights $w_{i:N}^{k-1}$.

(b) Perturb θ* through sampling θ** ~ *q*(*θ*|θ*>), where $q=N\left(\theta^{*},\sigma_{k-1}^{2}\right)$ or *q *= *U*(*a, b*). Here values of *a*,*b *depend on θ^* ^and $\sigma_{k-1}^{2}$.

(c) Generate simulations and calculate the error *∈* using the same steps as in 2(*b*) ~ (*d*).

(d) For each particle, assign weights

$$w_{i}^{k}=\frac{\pi \left(\theta_{i}^{k}\right)b_{k}\left(\theta_{k}^{i}\right)}{\sum_{j=1}^{N}w_{j}^{k-1}q\left(\theta_{j}^{k-1}|\theta_{j}^{k},\sigma_{k-1}\right)}.$$

(e) Determine the variance for the particles in the *k*-th iteration

$$\sigma_{k}=\sqrt{\mathrm{var}\left(\theta_{\left\{1:N\right\}}^{k}\right).}$$

An alternative approach is to generate simulations over the observation time period but compare the error to experimental data at each time point. The approach locates somewhere between ABC SMC algorithm [17] and the proposed Algorithm 1. which is presented below. For simplicity we do not give a detailed algorithm, but just provide the key steps 2.b) ~ 2.d) that are different from those in Algorithm 1.

ABC SLD algorithm 2

2.b) Generate data **Y ***B_k _*times using θ*.

2.c) For *m *= 1, · · ·, *B_k _*and *l *= 1,2, · · ·, *n*, calculate the value of discrepancy *d*(**X***_l_*, **Y***_ml_*) and test for

$$\left|X_{1}-Y_{ml}\right|\leq \alpha X_{l}.$$

If it is true, let *b_ml_*(θ*) = 0, otherwise it is one.

2.d) Calculate

$$\in =\overset{n}{\underset{l=1}{\sum }}\frac{1}{B_{k}}\overset{B_{k}}{\underset{m=1}{\sum }}b_{ml}\left(\theta^{*}\right).$$

## Competing interests

The authors declare that they have no competing interests.

## Authors' contributions

TT conceived and designed the study. QW and TT developed algorithms and carried out research. QW, KS and TT analyzed the data, interpreted the results and wrote the paper. All authors edited and approved the final version of the manuscript.

## Declarations

The publication costs for this article were funded by the corresponding author.

This article has been published as part of *BMC Bioinformatics *Volume 15 Supplement 12, 2014: Selected articles from the IEEE International Conference on Bioinformatics and Biomedicine (BIBM 2013): Bioinformatics. The full contents of the supplement are available online at http://www.biomedcentral.com/bmcbioinformatics/supplements/15/S12.

## References

[B1] Zhan C Yeung LF Parameter estimation in systems biology models using spline approximation BMC systems biology 2011 5 14 10.1186/1752-0509-5-14 21255466 PMC3750107

[B2] Kikuchi S Tominaga D Arita M Takahashi K Tomita M Dynamic modeling of genetic networks using genetic algorithm and S-system Bioinformatics 2003 19 5 643 650 10.1093/bioinformatics/btg027 12651723

[B3] Gadkar KG Gunawan R Doyle FJ Iterative approach to model identification of biological networks BMC bioinformatics 2005 6 155 10.1186/1471-2105-6-155 15967022 PMC1189077

[B4] Gonzalez OR Küper C Jung K Naval PC Mendoza E Parameter estimation using Simulated Annealing for S-system models of biochemical networks Bioinformatics 2007 23 4 480 486 10.1093/bioinformatics/btl522 17038344

[B5] Deng Z Tian T A continuous approach for inferring parameters in mathematical models of regulatory networks BMC bioinformatics 2014 15 256 10.1186/1471-2105-15-256 25070047 PMC4261783

[B6] Tian T Smith-Miles K Mathematical modelling of GATA-switching for regulating the differentiation of hematopoietic stem cell BMC bioinformatics 2014 8 S8 S8 10.1186/1752-0509-8-S1-S8 PMC4080254 24565335

[B7] Kirkpatrick S Gelatt CD Vecchi MP Optimization by Simulated Annealing Science 1983 220 4598 671 680 10.1126/science.220.4598.671 17813860

[B8] Mendes P Kell D Non-linear optimization of biochemical pathways: applications to metabolic engineering and parameter estimation Bioinformatics 1998 14 10 869 883 10.1093/bioinformatics/14.10.869 9927716

[B9] Srinivas M Patnaik LM Genetic algorithms: A survey Computer 1994 27 6 17 26

[B10] Ashyraliyev M Jaeger J Blom J Parameter estimation and determinability analysis applied to Drosophila gap gene circuits BMC Systems Biology 2008 2 83 10.1186/1752-0509-2-83 18817540 PMC2586632

[B11] Moles CG Mendes P Banga JR Parameter estimation in biochemical pathways: a comparison of global optimization methods Genome research 2003 13 11 2467 2474 10.1101/gr.1262503 14559783 PMC403766

[B12] Lall R Voit EO Parameter estimation in modulated, unbranched reaction chains within biochemical systems Computational biology and chemistry 2005 29 5 309 318 10.1016/j.compbiolchem.2005.08.001 16213792

[B13] Lillacci G Khammash M Parameter estimation and model selection in computational biology PLoS computational biology 2010 6 3 el000696 10.1371/journal.pcbi.1000696 PMC2832681 20221262

[B14] Goel G Chou IC Voit EO System estimation from metabolic time-series data Bioinformatics 2008 24 21 2505 2511 10.1093/bioinformatics/btn470 18772153 PMC2732280

[B15] Raj A van Oudenaarden A Nature, nurture, or chance: stochastic gene expression and its consequences Cell 2008 135 2 216 226 10.1016/j.cell.2008.09.050 18957198 PMC3118044

[B16] Wilkinson DJ Bayesian methods in bioinformatics and computational systems biology Briefings in, bioinformatics 2007 8 2 109 116 17430978 10.1093/bib/bbm007

[B17] Toni T Welch D Strelkowa N Ipsen A Stumpf MP Approximate Bayesian computation scheme for parameter inference and model selection in dynamical systems Journal of the Royal Society Interface 2009 6 31 187 202 10.1098/rsif.2008.0172 PMC2658655 19205079

[B18] Battogtokh D Asch DK Case ME Arnold J Schüttler HB An ensemble method for identifying regulatory circuits with special reference to the qa gene cluster of Neurospora crassa Proceedings of the National Academy of Sciences 2002 99 26 16904 16909 http://www.pnas.org/content/99/26/16904.abstract 10.1073/pnas.262658899 PMC139242 12477937

[B19] Sisson SA Fan Y Tanaka MM Sequential monte carlo without likelihoods Proceedings of the National Academy of Sciences 2007 104 6 1760 1765 10.1073/pnas.0607208104 PMC1794282 17264216

[B20] Beaumont MA Zhang W Balding DJ Approximate Bayesian Computation in Population Genetics Genetics 2002 162 4 2025 2035 12524368 10.1093/genetics/162.4.2025 PMC1462356

[B21] Marjoram P Molitor J Plagnol V Tavaré S Markov chain Monte Carlo without likelihoods Proceedings of the National Academy of Sciences 2003 100 26 15324 15328 10.1073/pnas.0306899100 PMC307566 14663152

[B22] Pritchard JK Seielstad MT Perez-Lezaun A Feldman MW Population growth of human Y chromosomes: a study of Y chromosome microsatellites Molecular Biology and Evolution 1999 16 12 1791 1798 10.1093/oxfordjournals.molbev.a026091 10605120

[B23] Fearnhead P Prangle D Constructing summary statistics for approximate Bayesian computation: semi-automatic approximate Bayesian computation Journal of the Royal Statistical Society: Series B (Statistical Methodology) 2012 74 3 419 474 10.1111/j.1467-9868.2011.01010.x

[B24] Marjoram P Tavaré S Modern computational approaches for analysing molecular genetic variation data Nature Reviews Genetics 2006 7 10 759 770 10.1038/nrg1961 16983372

[B25] Tanaka MM Francis AR Luciani F Sisson S Using approximate Bayesian computation to estimate tuberculosis transmission parameters from genotype data Genetics 2006 173 3 1511 1520 10.1534/genetics.106.055574 16624908 PMC1526704

[B26] Thornton K Andolfatto P Approximate Bayesian inference reveals evidence for a recent, severe bottleneck in a Netherlands population of Drosophila melanogaster Genetics 2006 172 3 1607 1619 16299396 10.1534/genetics.105.048223 PMC1456302

[B27] Picchini UL Inference for SDE models via Approximate Bayesian Computation Journal of Computational and Graphical Statistics in press 2014

[B28] Wu Q Smith-Miles K Tian T Approximate Bayesian computation for estimating rate constants in biochemical reaction systems Bioinformatics and Biomedicine (BIBM), 2013 IEEE International Conference on 2013 416 421

[B29] Daigle BJ Roh MK Petzold LR Niemi J Accelerated maximum likelihood parameter estimation for stochastic biochemical systems BMC bioinformatics 2012 13 68 10.1186/1471-2105-13-68 22548918 PMC3496601

[B30] Gillespie DT Exact stochastic simulation of coupled chemical reactions The journal of physical chemistry 1977 81 25 2340 2361 10.1021/j100540a008

[B31] Tian T Xu S Gao J Burrage K Simulated maximum likelihood method for estimating kinetic rates in gene expression Bioinformatics 2007 23 84 91 10.1093/bioinformatics/btl552 17068087

[B32] Wang Y Christley S Mjolsness E Xie X Parameter inference for discretely observed stochastic kinetic models using stochastic gradient descent BMC systems biology 2010 4 99 10.1186/1752-0509-4-99 20663171 PMC2914651

[B33] Golightly A Wilkinson DJ Bayesian inference for stochastic kinetic models using a diffusion approximation Biometrics 2005 61 3 781 788 10.1111/j.1541-0420.2005.00345.x 16135029

[B34] Reinker S Altman R Timmer J Parameter estimation in stochastic biochemical reactions IEE Proceedings-Systems Biology 2006 153 4 168 178 10.1049/ip-syb:20050105 16986618

[B35] Tian T Burrage K Binomial leap methods for simulating stochastic chemical kinetics The Journal of chemical physics 2004 121 21 10356 10364 10.1063/1.1810475 15549913

[B36] Pahle J Biochemical simulations: stochastic, approximate stochastic and hybrid approaches Briefings in bioinformatics 2009 10 53 64 19151097 10.1093/bib/bbn050 PMC2638628

[B37] Burrage K Tian T Burrage P A multi-scaled approach for simulating chemical reaction systems Progress in biophysics and molecular biology 2004 85 2 217 234 15142745 10.1016/j.pbiomolbio.2004.01.014

[B38] Boys RJ Wilkinson DJ Kirkwood TB Bayesian inference for a discretely observed stochastic kinetic model Statistics and Computing 2008 18 2 125 135 10.1007/s11222-007-9043-x

[B39] Golightly A Wilkinson DJ Bayesian parameter inference for stochastic biochemical network models using particle Markov chain Monte Carlo Interface Focus 2011 1 6 807 820 10.1098/rsfs.2011.0047 23226583 PMC3262293

[B40] Hurn AS Jeisman J Lindsay KA Seeing the wood for the trees: A critical evaluation of methods to estimate the parameters of stochastic differential equations Journal of Financial Econometrics 2007 5 3 390 455 10.1093/jjfinec/nbm009

[B41] Hurn A Lindsay K Estimating the parameters of stochastic differential equations Mathematics and computers in simulation 1999 48 4 373 384

